# Influence of Filament Winding Tension on the Deformation of Composite Flywheel Rotors with H-Shaped Hubs

**DOI:** 10.3390/polym14061155

**Published:** 2022-03-14

**Authors:** Xiaodong Chen, Yong Li, Dajun Huan, Hongquan Liu, Lisa Li, Yanrui Li

**Affiliations:** 1 College of Materials Science and Technology, Nanjing University of Aeronautics and Astronautics, Nanjing 210016, China; cxd4603@163.com (X.C.); huandj@nuaa.edu.cn (D.H.); liuhongquan@nuaa.edu.cn (H.L.); lls857762634@nuaa.edu.cn (L.L.); niyanrui@nuaa.edu.cn (Y.L.); 2 National Key Laboratory of Science and Technology on Helicopter Transmission, Nanjing University of Aeronautics and Astronautics, Nanjing 210016, China

**Keywords:** residual stress, process monitoring, flywheel rotors, filament wound composites, finite element analysis

## Abstract

The residual stress plays an important role in composite flywheel rotors composed of filament windings. The fiber tension during high-prestressed winding is the main source of the rotor deformation and residual stress of composite layers. In this study, the effect of the winding tension gradient on deformation was monitored in real-time. Two types of in-plane winding tension fluctuation methods were developed to investigate the effect of tension on deformation. Online and offline measurements were performed for the strain acquisition. A wireless strain instrument was used for online deformation monitoring and a laser scanner was used for the offline surface reconstruction. Additionally, different filament winding strategies were carried out to improve the efficiency of the winding tension by finite element analysis. The results indicated that the deviation between numerical and experimental results was within 8%. Based on the proposed numerical method, the influence of the in-plane and out-of-plane winding tension gradient distributions on the rotation process of the H-shaped rotor was analyzed. An in-plane winding strategy with variable tension was developed, which increased the initial failure speed by 160%.

## 1. Introduction

With the rapid development of electricity-related industries, the demand for energy storage has become more severe [1,2,3,4]. Flywheels are considered perfect commercial energy storage devices due to their low maintenance cost, long life cycle, high efficiency, freedom from a depth of discharge effects, environmental friendliness, wide operating temperature range, and ability to survive harsh conditions [5,6]. The rotor is the heart of a flywheel energy storage system. Energy storage and release are achieved by increasing and decreasing the rotor speed. The energy storage capacity is proportional to the rotor mass and proportional to the square of its rotational speed [7]. Therefore, finding a way to improve the rotor speed limit is highly beneficial for enhancing the energy storage efficiency of flywheels [8,9,10]. However, the failure risk increases with increasing speed. High-performance fiber-reinforced polymer (FRP) composite with outstanding specific strength and stiffness is widely used as an important engineering material. Thanks to its constantly decreasing raw material cost and rapidly developing processing technology, it has been applied in many engineering fields, such as FRP composite slab [11], structural reinforcement and repair in the field of traditional architecture [12], main load bearing components in the field of commercial aviation [13], and typical plates and pipes in the field of sporting goods [14]. Meanwhile, FRP composites are a perfect solution to the high-energy storage capacity flywheels [15,16,17].

The hoop strength of FRP composite rotors is sufficiently high, whereas the weak interlaminar strength limits their energy storage potential. Two-dimensional woven and 3D textile structure composite rotors have been proposed to enhance the radial strength [18,19,20]. However, their low fiber volume fraction and high manufacturing cost limit their wide application. The press-fitted multi-rim composite rotor not only alleviates the interlayer problem but also is economically friendly, is efficient, and has excellent design freedom. Ha [21] optimized the interlaminar interference and ply angles with strength ratios as the objective function and then proposed an optimal design scheme for hybrid rotors [9]. Kim [22] found that the number and sequence of materials along the radial direction were critical in maximizing the total stored energy of multi-rim rotors using a modified generalized plane strain assumption. Lee [23] proposed an online curing method for thick composite cylinders that could homogenize the radial temperature gradient and lower the residual stress levels caused by curing. Liu [24] equalized winding tensions to the residual stress in fibers based on the inverse iteration principle and proposed a process-induced theoretical model to calculate the residual stress. Zu [25] modelled the winding tension using the equivalent temperature cooling method to fully understand the winding tensions, curing times, and interactions between layers.

Reasonable hub designs are also vital for high-energy storage density flywheels. Kim et al. [26] proposed a dome-type hub to offset hoop strain. Zu et al. [27] studied the binding effect in the winding process of arch-shaped hubs using the finite element method. Most of the available studies have highlighted the theoretical calculation of cylindrical rotors with respect to different design variables [28,29,30,31,32]. However, there is generally support in flywheel rotors to transfer torque from the shaft. The hub in the flywheel rotor with high winding tension combined with online curing is then retained in the final product and there is no demolding or interference assembly procedure.

Filament wound composites have been proven to be an effective solution for flywheel rotors. The residual stress of flywheel rotors made of filament windings can significantly affect the burst speed during service, while the prestress is mainly determined by the winding tension. However, few researchers have considered the effects of metal hubs on the preloading process of composite rotors. Additionally, few studies paid attention to the mismatch between the design winding tensions and the residual stress caused by the complex relaxation in the manufacturing process. In this paper, the mismatch between the prestress caused by the winding tension and the residual stress measured in real time was studied through experimental and numerical analysis. H-shaped hubs were wrapped with FRP composites using different winding tensions. The process-induced rotor deformations were monitored by online wireless strain gauges and confirmed by offline reconstruction. A finite element-based model was used to study the influence of winding tensions on the prestressing effect of a mandrel and the subsequent failure process. The outcome of this study provides a tension design model considering the manufacturing process, which is helpful for the wide application of continuous fiber-reinforced thermosetting composites on high-quality standard rotary components.

## 2. Experimental study

### 2.1. Materials and Equipment

High-performance fibers used here included glass fiber (S6 supplied by Nanjing Fiberglass R&D Institute) and carbon fiber (T700SC, Toray, Japan and T800SC, Toray, Japan). The detailed performance parameters are shown in Table 1. E-8012 was used for the resin matrix, which was composed of two kinds of multifunctional epoxy resin and amine-curing agents with a tensile strength of 97 MPa. The initial curing temperature, peak curing temperature, and end curing temperature were 64 °C, 91 °C, and 123 °C, respectively. [33]. Aluminum 7075 (Al7075) was used as the hubs of flywheel rotors. Mechanical property tests were conducted on an electronic universal testing machine (SANS Inc., Shanghai, China).

### 2.2. Fabrication of Flywheel Rotor

**Figure 1:**
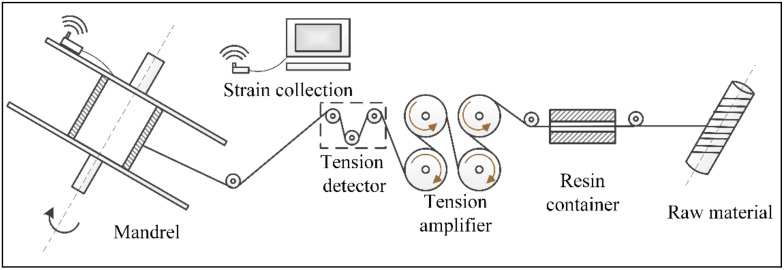
Schematic and equipment of winding system.

In the filament winding process, fiber tows were wound to the surface of the mandrel successively through a feeding shaft, a dipping tank, a tension amplifier, a tension detector, and a fiber guide nozzle (see Figure 1). The initial fiber tension was amplified by magnetic powder motors. The amplification coefficient was controlled by adjusting the motor torque through a controllable input current. The key point of tension amplification is to maintain the static friction force between the fiber bundle and the roller surface. Once relative slip occurs, the input current of the motor should be adjusted downwards in time. Otherwise, tension control accuracy will be affected and fiber damage will occur.

**Figure 2:**
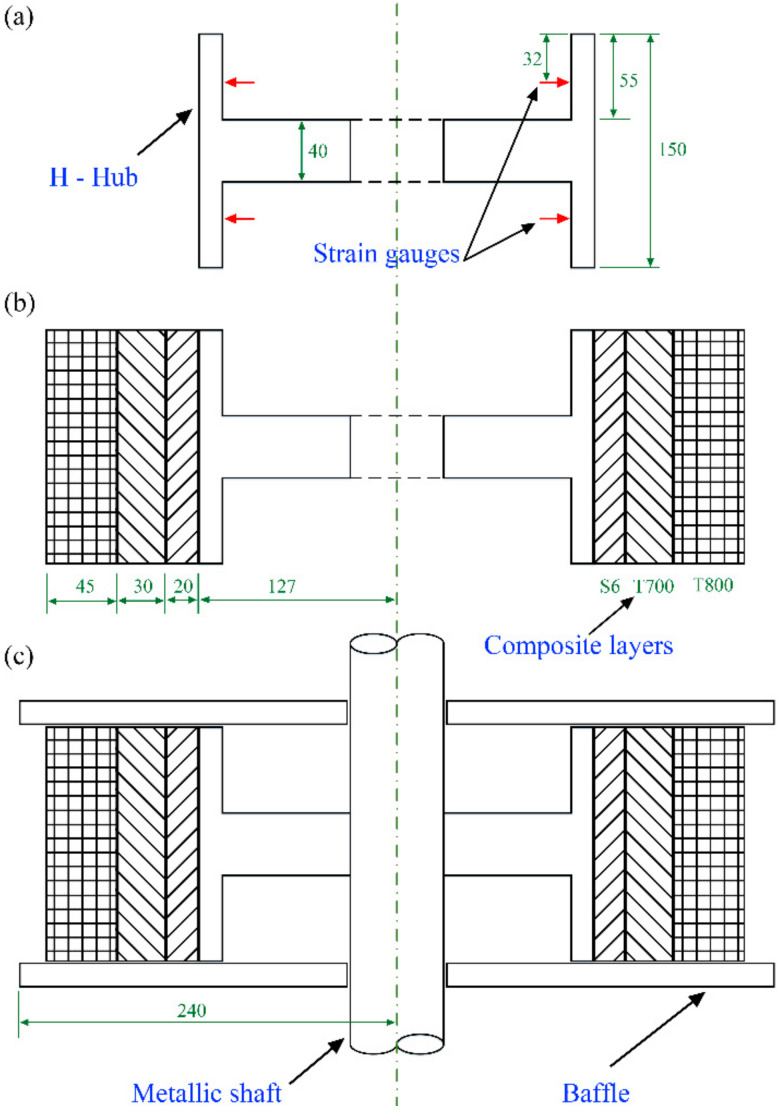
Diagram of flywheel rotor: (**a**) dimension of H-shaped hub and position of strain gauges; (**b**) dimension of composite layers; and (**c**) diagram of baffles.

The flywheel rotor with hybrid materials and the distribution of both inner glass fiber and outer carbon fiber can obtain higher energy storage density [22]. Composite layers were designed as GF-S6, CF-T700, and CF-T800 from the inside-out, and the thickness was 20 mm, 30 mm, and 45 mm, respectively (see Figure 2b). The geometric characteristics of the H-shaped hub and the strain acquisition position are shown in Figure 2a. There were four groups of strain gauges on both sides, which were evenly distributed on the concentric circles of the mandrel. Baffles were added to both sides of the H-shaped hub to prevent transverse movement of fiber during wet winding, as shown in Figure 2c. To study the influence of winding tensions on the deformation of H-shaped hubs, constant tension winding (CTW) and in-plane winding with variable tensions (IPWVT) were adopted. The purpose of winding variations in IPWVT was to reduce the preload of the unsupported part and prevent large plastic deformation.

The winding process should be stopped in advance when the deformation of rotors enters the plastic zone. The deformation of the mandrel causes the radial displacement of the wound composite layers, which leads to the relaxation of the fiber tension and weakens the prestressing effect of the fibers. Once the plastic deformation occurs, the deformation of the mandrel will be larger at the same level of the winding tension and the relaxation will also be amplified. This is not conducive to improving the energy storage density of the rotor. On the other hand, large mandrel deformation brings the risk of rotor size change. If the baffle stiffness is insufficient, the lateral dimensions of the rotor and subsequent parts’ assembly will be affected. 

### 2.3. Quantization of Deformations

The relationship between the deformation and the applied stress had been derived in our previous study [33].

(1) $$\varepsilon_{\theta }=\frac{2Fb^{2}}{E\left(a^{2}-b^{2}\right)}$$

where *ε_θ_* is the deformation of the mandrel; *F* is the winding tension, MPa; *E* is the modulus of the mandrel, MPa; and *a* and *b* is the inner and outer radius of the mandrel, mm. We can find a linear relationship between winding tension *F* and deformation *ε_θ_* according to the principle of elasticity. In this paper, the influence of the winding tension on the mandrel was quantitatively analyzed by deformation. Although the equation was established based on a cylindrical mandrel without a hub, the effect of the stress fluctuations can be effectively quantified by the deformations. Based on this assumption, the deformation of the inner mandrel was monitored by online and offline methods. Online strain collection adopted the DH3819 wireless strain gauge (Donghua Inc., Shang Hai, China) with a sampling frequency of 1 Hz. Offline strain acquisition was performed with the help of the ATOS Compact Scan (Gom Metrology GmbH, Brunswick, Germany). Additionally, it was calculated by the relative change of the reconstructed inner surface.

### 2.4. Experimental Results and Discussion

#### 2.4.1. Results of Mechanical Properties and Microstructure

**Figure 3:**
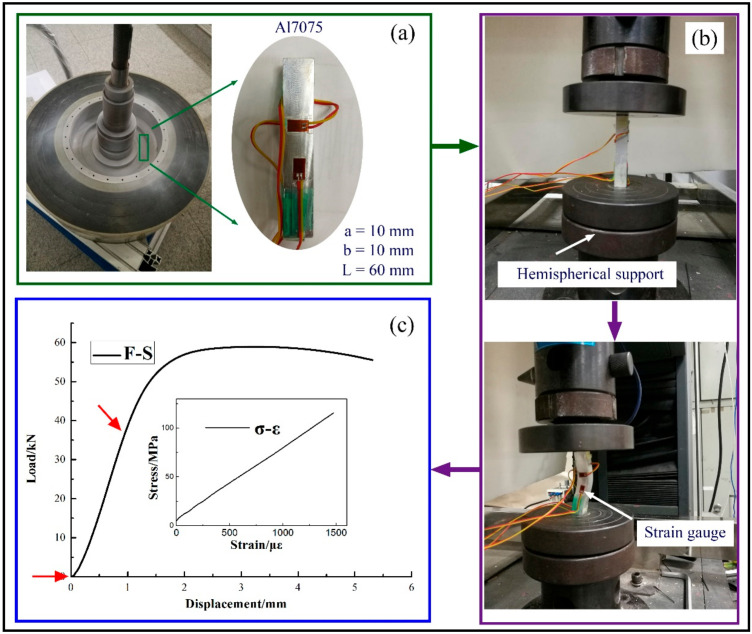
Characterization of mandrel material properties: (**a**) sample location and dimension; (**b**) test procedure; and (**c**) test results.

The typical compressive stress–strain curve of the Al7075 is shown in Figure 3. The compressive strength of the Al7075 was 578.32 ± 21.39 MPa, the yield strength was 480.28 ± 17.00 MPa, the elastic modulus was 74.07 ± 1.00 GPa, and the Poisson’s ratio was 0.33 according to the standard test methods of the compression testing of metallic materials at room temperature (GB/T 7314-2017). It was demonstrated that the hubs were all in the elastic stage within a strain of −6480 × 10^−6^. The measured elastic parameters of the mandrel laid the foundation for deformation monitoring.

**Figure 4:**
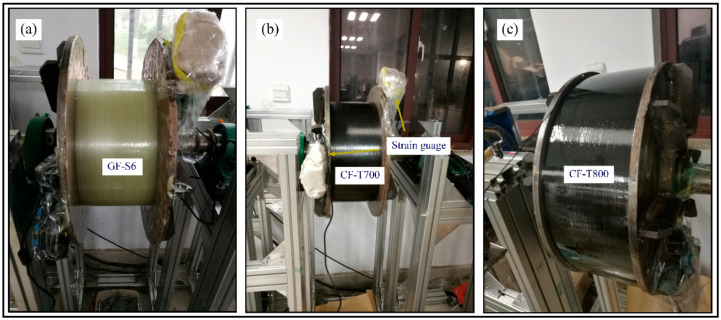
Winding process of: (**a**) glass fiber GF-S6, (**b**) carbon fiber T700, and (**c**) carbon fiber T800.

**Figure 5:**
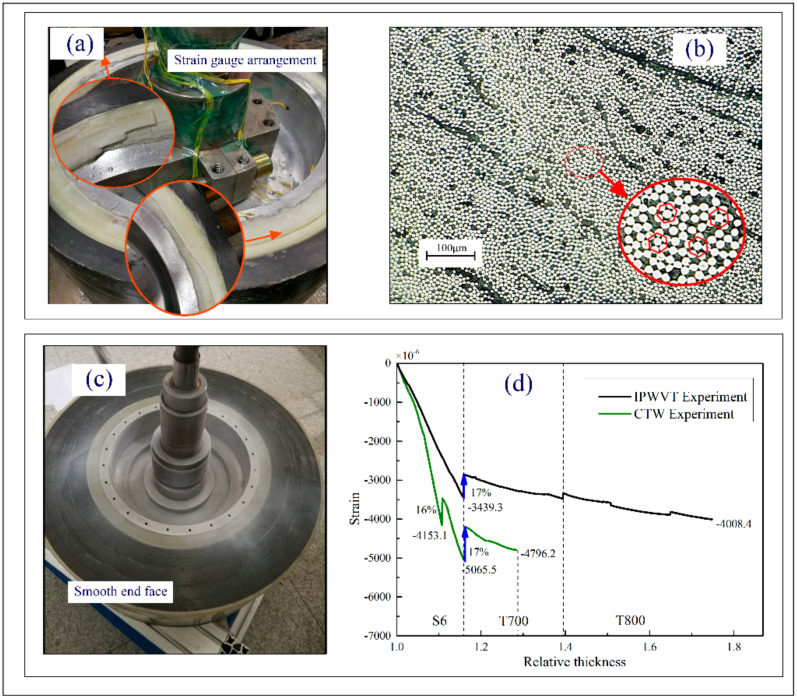
(**a**) Morphology of CTW and strain gauge arrangement; (**b**) metallographic picture; (**c**) morphology of IPWVT; and (**d**) results of online strain with respect to relative thickness.

Figure 4 showed a schematic of the winding processes. There was an external infrared lamp mounted for the rapid cure of the resin matrix. The strain gauges were wrapped with glass fiber cloth to protect the instrument from the influence of temperature. It can be seen from the microscopic metallographic pictures (see Figure 5b) that the structure of the composite layers was compact and the fiber arrangement was mostly hexagonal. Almost all single fibers were in contact with each other, indicating that the resin matrix was effectively extruded. When the external load was transferred from the outermost layer to the inner layers, the fiber mesh bore most of the deformation [34].

#### 2.4.2. Results of Online and Offline Deformation Monitoring

The detection positions of the strain gauges were symmetric (see Figure 5a). The online deformation detection results are shown in Figure 5d. The relative thickness *λ* was defined to describe the thickness of the winding layer,

(2) $$\lambda =r_{i}/r_{b}$$

where *r_i_* is the outer radius of the winding layers and *r_b_* is the outer radius of the metal hubs. The strain of the composite flywheel rotors formed by the two winding tension systems went up with the winding process, indicating that the winding tension of the external fibers can be effectively transferred to the mandrel. However, the effect of outer fiber tension on the mandrel decreased with increasing thickness, which may have been caused by resin matrix relaxation and fiber mesh deformation. The slope of the strain curve of the mandrel increased significantly at the initial stage of winding, indicating that the cross-linking of the polymer matrix froze the deformation of the inner fiber mesh and resin flow, thus effectively pre-tightening the mandrel. The slope the of strain curve of the glass fiber layers was significantly higher than that of the carbon fiber in both winding processes. The prestressing effect of glass fibers on the inner layers was better than that of the carbon fiber, which was consistent with the conclusion drawn in the literature [9,26,32,35].

The slope of the strain curve of the flywheel rotor with CTW was higher than that with IPWVT. However, the slope of the strain curve at the edge of the rotor of CTW was sharp, leading to the yield of the hub. From the side picture after the removal of the baffle (see Figure 5a), it can be found that part of the glass fiber layers was crushed and there was a fiber delamination failure in the symmetrical position. In the winding process, the axial prestress of the winding layers increased with the number of winding layers. The transverse deformation of CTW was more severe than that of IPWVT. Online deformation monitoring indicated that the mandrel yielded during the late CTW period, which made it difficult to guarantee the transverse dimension. After curing, these deformations were preserved by the cross-linked resin matrix to form the prestress in the rotors. With the removal of the baffles, the transverse boundary constraint was released and the load formed by the transverse deformation of the CTW parts exceeded the interlaminar bonding strength of the composite material, resulting in transverse delamination failure. The failure locations of the specimen were symmetrically distributed, which also accorded with the symmetrical characteristics of the uniform distribution of the fiber preload in the circumferential direction. The winding layers piled up at both ends provided the additional bending moment of the mandrel, resulting in the abnormal slope of the strain curve and fiber collapse of the end face. In contrast, for the rotor with IPWVT, the slope of the strain curve increased relatively gently and the plastic deformation did not happen even at the end of the winding process. After winding, the end face was smooth and flat (see Figure 5c), and boundaries of resin-rich areas left by multiple curing processes could also be observed clearly.

In both CTW and IPWVT processes, it could be found that there was a large stress relaxation phenomenon after every multiple curing processes. The temperature change had a significant influence on the deformation and the relaxation of the glass fiber layers after curing reached approximately 17%. However, the change in winding tension had little influence on stress relaxation. This indicates that the preload difference formed in the winding process is retained in proportion to the cured product. As the number of winding layers increased, the heat input from the infrared lamp was surrounded by glass fibers and was difficult to transfer outward, leading to a gradual increase in the deformation of the mandrel. It can be seen from Figure 5d that the slope of the strain curve in the initial stage increased faster. When the temperature change in the mandrel was in equilibrium with the external heat source input, the influence of thermal expansion was no longer dominant. Winding tension became the key factor for mandrel deformation. It can be seen from Figure 5d that the growth rate of the mandrel strain of the IPWVT decreased significantly. However, the strain increased rapidly in the first stage of the CTW and the slope of the strain curve did not decrease even at the end of the winding.

**Figure 6:**
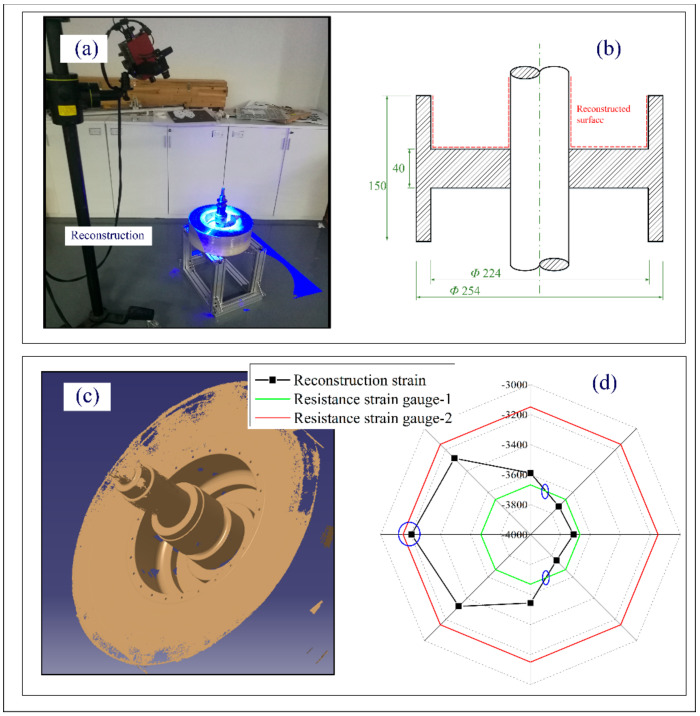
Offline strain collection: (**a**) reconstruction process; (**b**) reconstruction scheme; (**c**) reconstruction surface; and (**d**) comparison of online and offline results.

The offline deformation reconstruction process is shown in Figure 6. First, we sprayed the developer uniformly on the surface of interest, as shown in Figure 6b. Then, the ATOS Compact Scan was used to perform the collection of the point clouds of the inner surface, as shown in Figure 6a. According to the collected discrete point clouds, the fitting of the inner surface was completed with the assistance of CATIA, as shown in Figure 6c. Finally, the deformation of the mandrel was calculated based on the geometric morphology difference of the inner surface before and after winding, as shown in Figure 6d. The final deformation results of strain monitoring and reconstruction are shown in Figure 6d. The red and green lines in the figure represent the deformation detected by the strain gauge, which was −3149.5 and −3669.2, respectively. The black line is the strain value calculated after reconstruction. The corresponding strain values were calculated at eight equally divided positions on the concentric circle where the minimum strain was −3205.9 and the maximum strain was −3755.6. The strain gauge monitoring values showed a maximum deviation of 14.2%, while for the reconstructed strain, the maximum deviation reached 14.6%. There were two intersection points and a relatively close value between the reconstructed strain and the strain gauge monitoring value, indicating that the test results of these positions are close, confirming the rationality of the two test methods. Interestingly, the deformation of the reconstructed surface deviated along the circumferential direction with two peak deviations of 14%, indicating that the deformation no longer kept a regular cylinder shape, which may bring hidden trouble to the subsequent dynamic balance.

## 3. Finite Element Analysis (FEA)

The model established in this paper was based on the commercial software ABAQUS, in which the simulation during the winding process used a comparison of the model change and the realization of the life and death unit technology in ABAQUS. According to the results of the winding experiments, the residual stress of CTW and IPWVT were quite different. The influence of winding tension fluctuations on rotor deformations was further discussed by FEA.

### 3.1. Finite Element Model and Boundary Conditions

**Figure 7:**
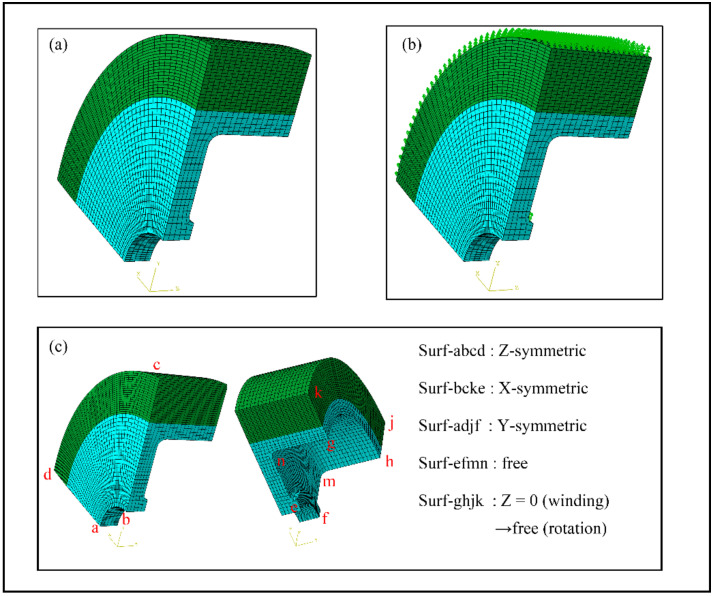
Modeling of flywheel rotor: (**a**) meshing (**b**); centrifugal load; and (**c**) boundary conditions.

To simulate the influence of winding tensions on the rotor deformation, a 1/8 model was established (see Figure 7). A solid 6188 three-dimensional element (C3D8R) was used to model the Al 7075 mandrel. The composite layers were simulated with the three-dimensional element C3D8I and the total element was 12,920. The composite layers were described by orphan mesh-based elements offset from the surface of the mandrel. A surface-to-surface contact between the mandrel and the composite was added with a frictional coefficient of 0.15. The boundary conditions and loading distribution are shown in Figure 7. Symmetric boundary conditions were applied to the surface of Surf-abcd, Surf-bcke, and Surf-adjf. The axial end Surf-ghjk was set as a fixed boundary condition in the winding process and switched to a free boundary condition in the rotation process because baffles would be removed after curing. The temperature parameters method and the model change function of ABAQUS were used to simulate the winding process. Centrifugal loads were applied in a quasi-static manner by rotational body force. In the process of rotation, the failure criterion of the metal mandrel adopted the Mises criterion, while the failure criterion of composite materials referred to the improved Harshin criterion [36].

### 3.2. Modeling of Fiber Tension

**Figure 8:**
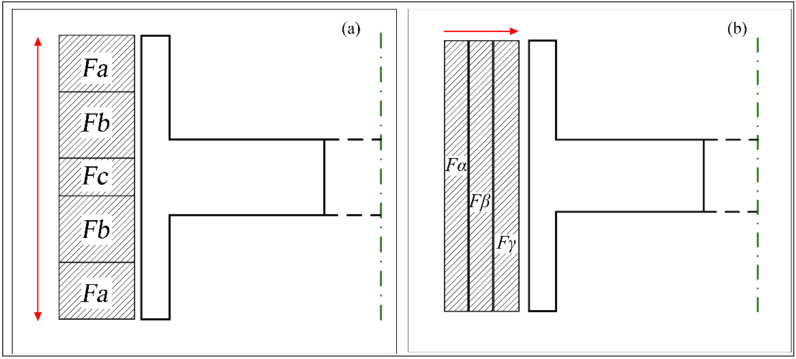
Winding tension distribution diagram: (**a**) IPWVT and (**b**) OPWVT.

Three types of winding tension rules were adopted, which were constant tension winding (CTW), in-plane variable tension winding (IPWVT), and out-of-plane variable tension winding (OPWVT). For IPWVT, the winding area was divided into five symmetric sub-units along the axial direction, as shown in Figure 8a. For OPWVT, the winding area was divided into three sub-units along the radial direction, as shown in Figure 8b. The winding tension parameters are shown in Table 2. Only the glass fiber was used here to discuss the influence of winding tension on the rotor deformation in the winding process and the subsequent rotation process. The composite thickness was set at 40 mm. When the fiber tension of S6 was greater than 330 MPa, fiber damage would occur after the tension amplifier even if the fibers were fully infiltrated by the resin, as obtained in our previous experiments. Therefore, the maximum tension of the winding was determined as 300 MPa.

### 3.3. Failure Criterion and Degradation Model

The flywheel rotor was mainly composed of a metal hub and composite layers. The elastic parameters of the materials used in the model are shown in Table 3 and Table 4. The structural failure mode was divided into three types: yield failure of a metal hub, interface separation failure, and progressive failure of composite layers.

The Mises criterion was used to determine the failure of metal materials, expressed as follows

(3) $$F_{1}=\frac{{\left(\sigma_{1}-\sigma_{2}\right)}^{2}+{\left(\sigma_{2}-\sigma_{3}\right)}^{2}+{\left(\sigma_{3}-\sigma_{1}\right)}^{2}}{2K^{2}}>1$$

Contact properties were inserted between composite layers and metal liners so the output contact pressure could be used directly to determine whether the interface was separated.

(4) $$F_{2}=CPRESS=0$$

A modified Hashin failure criterion was used to predict the damage initiation of composites. The fiber damage initiation index is expressed by the criterion

(5) $$F_{31}^{t}=\sqrt{{\left(\frac{\sigma_{11}}{Xt}\right)}^{2}+{\left(\frac{\sigma_{12}}{S_{12}}\right)}^{2}+{\left(\frac{\sigma_{13}}{S_{13}}\right)}^{2}}>1,\left(\sigma_{11}>0\right)$$

(6) $$F_{31}^{c}=\sqrt{{\left(\frac{\sigma_{11}}{Xc}\right)}^{2}}>1,\left(\sigma_{11}<0\right)$$

The matrix damage initiation index is expressed by the criterion

(7) $$F_{32}^{t}=\sqrt{{\left(\frac{\sigma_{22}}{Yt}\right)}^{2}+{\left(\frac{\sigma_{12}}{S_{12}}\right)}^{2}+{\left(\frac{\sigma_{23}}{S_{23}}\right)}^{2}}>1,\left(\sigma_{11}+\sigma_{33}>0\right)$$

(8) $$F_{32}^{c}=\sqrt{{\left(\frac{\sigma_{22}}{Yc}\right)}^{2}+{\left(\frac{\sigma_{12}}{S_{12}}\right)}^{2}+{\left(\frac{\sigma_{23}}{S_{23}}\right)}^{2}}>1,\left(\sigma_{11}+\sigma_{33}<0\right)$$

The shear damage of the fiber and matrix is expressed as follows:

(9) $$F_{33}^{}=\sqrt{{\left(\frac{\sigma_{11}}{Xc}\right)}^{2}+{\left(\frac{\sigma_{12}}{S_{12}}\right)}^{2}+{\left(\frac{\sigma_{13}}{S_{13}}\right)}^{2}}>1,\left(\sigma_{11}<0\right)$$

The out-of-plane delamination damage is expressed as follows:

(10) $$F_{34}^{t}=\sqrt{{\left(\frac{\sigma_{33}}{Zt}\right)}^{2}+{\left(\frac{\sigma_{13}}{S_{13}}\right)}^{2}+{\left(\frac{\sigma_{23}}{S_{23}}\right)}^{2}}>1,\left(\sigma_{33}>0\right)$$

(11) $$F_{34}^{c}=\sqrt{{\left(\frac{\sigma_{33}}{Zc}\right)}^{2}+{\left(\frac{\sigma_{13}}{S_{13}}\right)}^{2}+{\left(\frac{\sigma_{23}}{S_{23}}\right)}^{2}}>1,\left(\sigma_{33}<0\right)$$

where $\sigma_{i}\left(i=1,2,3\right)$ is the principal stress of metal materials and *K* equals the yield strength of AL7075. $\sigma_{ij}\left(i=1,2,3;j=1,2,3\right)$ is the stress component of the composite materials. It is acceptable to have a small amount of plastic deformation in the winding stage but when the plastic deformation occurs (*F*_1_ > 1) in the service process, the structure will fail. Once the pressure between the metal hub and composite layers is equal to zero (*F*_2_ = 0), the structure will also fail. However, the failure of composite layers is a gradual process. When an element fails, its bearing capacity decreases, but the surrounding elements still have a certain bearing capacity. At the same time, the ability of the structure to resist deformation decreases correspondingly, which is called stiffness softening [37]. The definition of failure degradation coefficients is shown in Equations (12) and (13), and the specific parameters are shown in Table 5.

(12) $$\begin{array}{ccc} E_{1}^{d}=d_{1}E_{1} & E_{2}^{d}=d_{2}E_{2} & E_{3}^{d}=d_{3}E_{3}\\ G_{12}^{d}=d_{4}G_{12} & G_{13}^{d}=d_{5}G_{13} & G_{23}^{d}=d_{6}G_{23} \end{array}$$

(13) $${\left[C_{ij}^{d}\right]}^{-1}=\left[S_{ij}^{d}\right]=\left[\begin{array}{cccccc} S_{11}/d_{1} & S_{12} & S_{13} & & & \\ S_{12} & S_{22}/d_{2} & S_{23} & & & \\ S_{13} & S_{23} & S_{33}/d_{3} & & & \\ & & & S_{44}/d_{6} & & \\ & & & & S_{55}/d_{5} & \\ & & & & & S_{66}/d_{4} \end{array}\right]$$

### 3.4. Numerical Results and Discussion

#### 3.4.1. Model Validation

**Figure 9:**
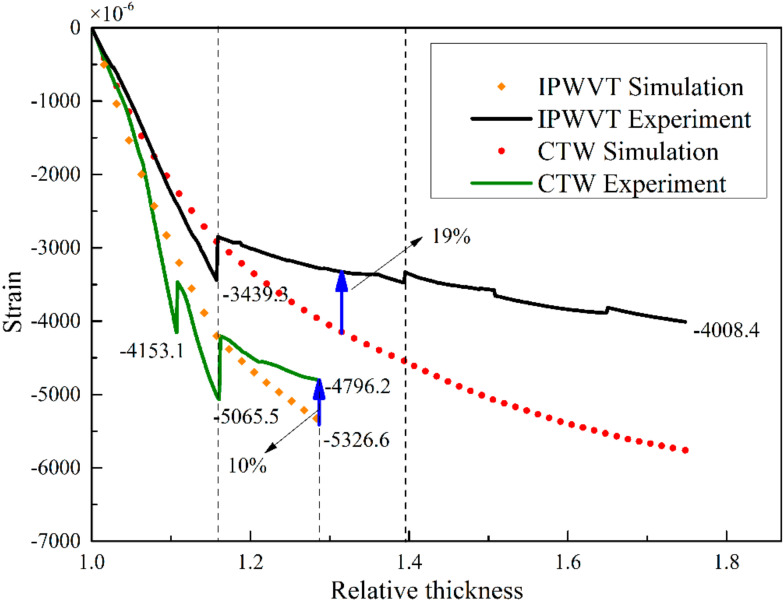
Comparison between online strain monitoring results and simulation results.

The comparison between the simulation results and the experimental results is shown in Figure 9. During the winding process, the deformation of the mandrel increased rapidly with the number of winding layers and then decreased suddenly after each curing process. In the initial stage of winding, the deformation decreased by 17% after curing regardless of winding tension variation, indicating that the deformation of the mandrel caused by temperature and that caused by winding tension are two independent sources. The residual deformation after curing was very close to the simulated strain and the difference was within 8%, indicating that the model proposed in this paper can well predict the residual deformation at the initial winding stage. However, when winding to the carbon fiber stage, the difference between the experimental and simulated strain became larger. The results of different winding tension confirmed this phenomenon. When the relative thickness *λ* reached about 1.3, the simulated strain of CTW was 10% higher and the simulated strain of IPWVT was almost 19% higher. This indicates that the model proposed in this paper cannot accurately simulate the deformation of mandrel when the relative thickness *λ* is greater than 1.3. When *λ* was less than 1.3, the experimental and simulation results of the residual strain were in good agreement; it may be the relaxation caused by the flow of resin. With the increase of winding thickness, although the influence of the thermal effect on deformation gradually decreased, the loss of the resin matrix influenced the deformation. The uncured resin in the inner layers could not restrain the deformation of the fibers in the accumulated high-stress area, thus the stress in the fibers relaxed gradually after each winding of the outer layers. However, it was difficult to simulate the resin extrusion process in the numerical model, thus deviation gradually increased after *λ* was greater than 1.3.

#### 3.4.2. Effect of Winding Tension on Failure Process

**Figure 10:**
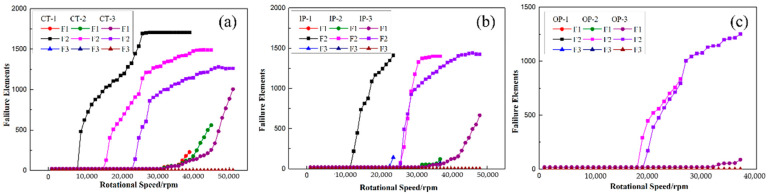
Element failure counts in FEA: (**a**) CTW cases; (**b**) IPWVT cases; and (**c**) OPWVT cases.

The number of failure elements of the rotors by different winding tensions was obtained by using the failure criteria proposed in this paper. It can be seen from Figure 10 that the main failure form of rotors in this paper was the separation of the interface between the metal hubs and composites with rising rotation speed, followed by the failure of the metal hub, and the failure of the composites occurred at the latest and least speed. This indicated that the prestress of composite layers was not enough to protect the mandrel from separation. Increasing the winding tension or the number of winding layers may be the possible solution. Considering that the winding tension proposed in this paper was close to the tension limit available for the wet winding of such fibers, increasing the number of winding layers may improve the interface failure before the failure of metal materials and composites. The interface separation of rotors by CT-1 has appeared at 10,000 rpm and the initial failure speed can be increased by 160% to more than 26,000 rpm by IPWVT. Additionally, the number of failure elements of the IP-2 rotor increased rapidly to the equilibrium value in a short speed range, indicating that its failure occurs suddenly and the difference of the failure strength at each position is small.

**Figure 11:**
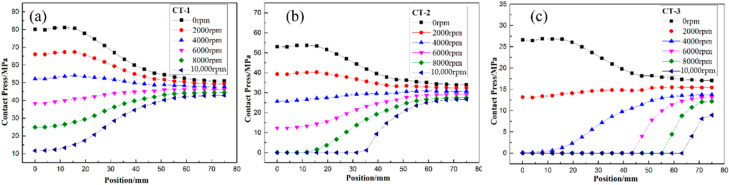
The distribution of contact stress of CTW rotor with winding tension of (**a**) CT-1 case; (**b**) CT-2 case; and (**c**) CT-3 case.

**Figure 12:**
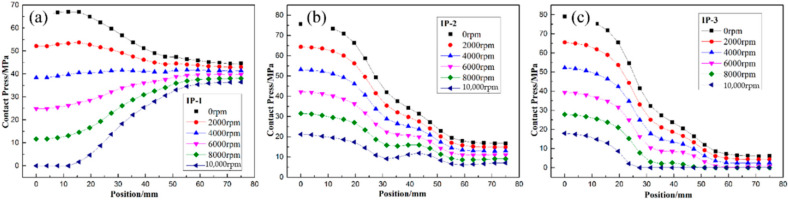
The distribution of contact stress of IPWVT rotor with winding tension of (**a**) IP-1 case; (**b**) IP-2 case; and (**c**) IP-3 case.

**Figure 13:**
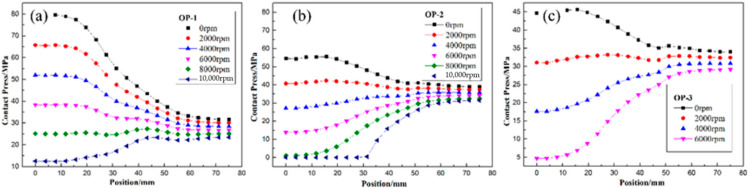
The distribution of contact stress of OPWVT rotor with winding tension of (**a**) OP-1 case; (**b**) OP-2 case; and (**c**) OP-3 case.

Furthermore, Figure 11, Figure 12 and Figure 13 illustrate the failure process of contact elements, where the position of 0 mm represents the middle of the H-hub and 75 mm represents the edge of the H-hub. In CTW with the same winding tension, the residual stress in the middle position with support was higher than that in the edge position. In IPWVT, the residual stress gradient at the middle and edge positions was the largest. With the rise of rotational speed, the interface separation first appeared in the middle position of the CTW rotors, indicating that the separation tendency of the metal with higher density and the composites in the middle position under a centrifugal load is smaller. However, in IPWVT rotors, with the increase of the prestress gradient, the separation area gradually draws closer to the edge of the rotor. For the IP-2 case, the initial interface failure speed reached 12,700 rpm, while this speed of the IP-1 was 11,100 rpm (Figure 10b). For the IP-1 case, the initial failure position was above the middle of the hub, whereas for the IP-2 and IP-3 cases, the failure position was above the edge of the hub (Figure 12). Compared with the CTW and OPWVT rotors, the interface stress distribution of IPWVT rotors was more uniform when a failure occurred. For OPWVT rotors, the initial interface failure speeds were all below 10,000 rpm and the first interface failure still occurred in the middle position above the hub (Figure 13). For the OP-1 case, the initial failure speed was close to 10,000 rpm, while that of the OP-3 case was only 6500 rpm. 

As can be seen from the differences in contact pressures of the rotors with rotational speeds under several winding tension schemes, the contact pressure at the lateral edge position increased with rotational speed while the contact pressure at the middle position with support decreased. A reasonable tension design can control the failure position of the interface in the most favorable position and also can realize the design of an equal strength at a specific speed so as to maximize the utilization rate of raw materials.

## 4. Conclusions

A comparison experiment was conducted to understand the influence of in-plane winding tension fluctuations on the H-hub deformation and a finite element model was proposed to further investigate both the influence of out-of-plane winding tension variations and their failure processes. The online and offline strain results confirm the viability of the proposed FEA model. The main conclusions of this study are summarized below.

(1) The compression properties of the elastic range of the rotor was within −6480 × 10^−6^ and it was effective to use the rotor deformation to characterize the composite deformation in this range.
(2) The constant winding tension caused a large deformation at the edge of the H-hub, leading to the plastic deformation of the hub, while the in-plane winding with variable tension prestressed the mandrel more evenly in the axial direction.
(3) When the relative thickness *λ* is greater than 1.3, the influence of the outer winding layer on the deformation of the mandrel gradually decreases. The subsequent curing process results in a relaxation of more than 17%, which is insensitive to the winding tension.
(4) Interface delamination failure comes first, followed by hub failure and composite failure, for a rotor with a relative thickness of 1.3. The rotor failure speed of IP-2 was 160% higher than that of CT-1 and reached 26,000 rpm.
(5) The beneficial residual stress in filament-wound flywheel rotors was successfully introduced by different fiber tensions, which could effectively improve the failure speed of flywheels. However, due to the viscoelastic characteristics of resin matrix, the effective storage time of this beneficial manufacturing stress needs to be further studied, which is valuable for the long-term performance of rotors.

## Figures and Tables

**Table 1:** Properties of raw materials.

Properties	S6	T700SC	T800SC	Unit
Tensile strength	4800	4900	5880	MPa
Tensile modulus	95	230	294	GPa
Strain at failure	5.7	2.1	2.0	%
Density	2.53	1.80	1.80	g/cm^3^
Yield	800	800	1030	g/km
CTE	3.00	−0.38	−0.40	×10^−6^/°C

**Table 2:** Winding tension design scheme.

Winding Rule	Case	Fa/MPa	Fb/MPa	Fc/MPa	Fα/MPa	Fβ/MPa	Fγ/MPa
OPWVT	OP-1	--	--	--	192	240	300
	OP-2	--	--	--	108	180	300
	OP-3	--	--	--	48	120	300
IPWVT	IP-1	300	240	192	--	--	--
	IP-2	300	180	108	--	--	--
	IP-3	300	120	48	--	--	--
CTW	CT-1	300	300	300	300	300	300
	CT-2	200	200	200	200	200	200
	CT-3	100	100	100	100	100	100

**Table 3:** Elastic parameters of Al7075.

Parameters	Symbol	Value	Unit
Modulus	*E*	74.07	GPa
Passion ratio	*v*	0.33	
Density	*ρ*	2.3	g/cm^−3^
Yield strength	*σ_s_*	480	MPa

**Table 4:** Mechanical properties of S6/epoxy composites.

Parameters	Symbol	Value	Unit
Longitudinal modulus	*E*_1_	67.46	GPa
Transverse modulus	*E*_2_ = *E*_3_	7	GPa
In-plane Poisson’s ratio	*v*_12_ *= v*_13_	0.29	
Transverse Poisson’s ratio	*v*_23_	0.34	
In-plane shear modulus	*G*_12_ = *G*_13_	4.14	GPa
Out-of-plane shear modulus	*G*_23_	3.4	MPa
Density	*Ρ*	1.8	g/cm^−3^
Longitudinal tensile strength	*Xt*	1660	MPa
Longitudinal compressive strength	*Xc*	610	MPa
Transverse tensile strength	*Yt*	34	MPa
Transverse compressive strength	*Yc = Zc*	118	MPa
Interlaminar tensile strength	*Zt*	15	MPa
In-plane shear strength	*S*_12_ *= S*_13_	94	MPa
Out-of-plane shear strength	*S*_23_	40	MPa

**Table 5:** Material stiffness degradation coefficients.

Composite Failure Mode (*F*_3_)	Coefficient					
	*E*_1_	*E*_2_	*E*_3_	*G*_12_	*G*_13_	*G*_23_
	*d*_1_	*d*_2_	*d*_3_	*d*_4_	*d*_5_	*d*_6_
Fiber damage (*F*_31_)	0.01	0.01	0.01	0.01	0.01	0.01
Matrix damage (*F*_32_)	1	0.3	1	1	1	0.01
Shear damage (*F*_33_)	1	1	1	0.01	1	1
Interlaminar damage (*F*_34_)	1	1	0.01	1	0.01	1

## Data Availability

The data presented in this study are available upon request from the corresponding author.
